# Analysis of questioning technique during classes in medical education

**DOI:** 10.1186/1472-6920-12-39

**Published:** 2012-06-12

**Authors:** Young Hye Cho, Sang Yeoup Lee, Dong Wook Jeong, Sun Ju Im, Eun Jung Choi, Sun Hee Lee, Sun Yong Baek, Yun Jin Kim, Jeong Gyu Lee, Yu Hyone Yi, Mi Jin Bae, So Jung Yune

**Affiliations:** 1 Family Medicine Clinic and Research Institute of Convergence of Biomedical Science and Technology, Pusan National University Yangsan Hospital, Yangsan, Gyeongsangnam-do, Republic of Korea; 2 Medical Education Unit and Medical Research Institute, Pusan National University, School of Medicine, Beomeo-ri Mulgeum-eup, Yangsan, Gyeongsangnam-do, 626-870, South Korea; 3 Department of Family Medicine, Pusan National University Hospital, Busan, South Korea; 4 Center for Teaching and Learning, Pusan National University, Busan, South Korea

**Keywords:** Questioning, Lecture, Medical education, Wait‒time, Faculty, Perception, Reality

## Abstract

**Background:**

Questioning is one of the essential techniques used by lecturers to make lectures more interactive and effective. This study surveyed the perception of questioning techniques by medical school faculty members and analyzed how the questioning technique is used in actual classes.

**Methods:**

Data on the perceptions of the questioning skills used during lectures was collected using a self‒questionnaire for faculty members (N = 33) during the second semester of 2008. The questionnaire consisted of 18 items covering the awareness and characteristics of questioning skills. Recorded video tapes were used to observe the faculty members’ questioning skills.

**Results:**

Most faculty members regarded the questioning technique during classes as being important and expected positive outcomes in terms of the students’ participation in class, concentration in class and understanding of the class contents. In the 99 classes analyzed, the median number of questions per class was 1 (0–29). Among them, 40 classes (40.4 %) did not use questioning techniques. The frequency of questioning per lecture was similar regardless of the faculty members’ perception. On the other hand, the faculty members perceived that their usual wait time after question was approximately 10 seconds compared to only 2.5 seconds measured from video analysis. More lecture‒experienced faculty members tended to ask more questions in class.

**Conclusions:**

There were some discrepancies regarding the questioning technique between the faculty members’ perceptions and reality, even though they had positive opinions of the technique. The questioning skills during a lecture need to be emphasized to faculty members.

## Background

Questioning techniques have long been used as the most common and effective teaching method, and studies have demonstrated that questions have an important effect on academic achievement for students [1,2]. Despite the importance of questioning, however, the use of questioning techniques during classes is very low in universities. A study that surveyed typical university class patterns reported that faculty members use only 4 % of the total class time for posing questions to students. According to the report, even if faculty members ask questions, they do not receive an answer from the students approximately 30 % of the time, and the faculty members give the answer instead of waiting for the students’ answer [3].

Tobin [4] said that waiting for at least 3–5 seconds after posing a question has a positive effect on the students’ achievement and the faculty members’ teaching performance. Other studies also reported that an extended waiting time after questioning increases the opportunities for the students to answer [5]. According to previous reports, however, the wait time after questioning until the students’ response in a university class was less than 3 seconds on average [6].

When a question is asked, the students analyze, combine or evaluate many pieces of information to answer the question correctly [7-10]. Therefore, the questioning technique is believed to have an important effect on students’ learning, particularly in medical schools that give high‒level education. On the other hand, there are few reports on the questioning technique in medical education. A previous pilot study reported that most medical faculty members perceived a usual wait-time of between 6 to 10 seconds, whereas the average wait time was actually only 0.6 seconds [11]. This wait time was not examined not by video recording analysis but by observations of the appointed student during lecture. Phillips and Duke [8] performed a comparative design to examine the questioning skills of 14 clinical teachers and 14 preceptors who had been involved in teaching undergraduate nursing students.

The data showed that clinical teachers had more experience in teaching and asked more questions at a higher cognitive level.

Therefore, this study was conducted to survey the perception of the questioning technique by medical school faculty members and examine how the questioning technique is used in actual classes by analyzing videos recorded during classes. The hypothesis was that there would be differences between the expected wait time and actual wait time. In addition, it was assumed that the faculty members’ position representing their years of experience in education affected the questioning technique in medical class. This study was carried out to answer the following research questions:

1. What is the medical school faculty member’s perception of the questioning technique?

2. What are the actual questioning behaviors in medical class of the faculty members by analyzing the video record?

3. Are there any differences between the expected wait time and actual wait time?

4. Does the faculty position affect the questioning technique in medical class?

## Methods

### Participants sampling

The study participants were 40 faculty members who had been lecturing to second year medical students from September 8 to November 14, 2008 at a medical school in South Korea.

### Instruments

The survey instruments included a questionnaire and observation record sheet according to the data collection method. The questionnaire was largely divided into the demographic characteristics and his/her opinions on the questioning technique of participants. The demographic characteristics (gender, age, organization, position, and teaching experience) were obtained from the answers to 5 questions. In addition, 10 questions focused on the questioning technique including the importance of the questioning technique, planning for questioning, mean number of questions, questioning time, question type, intended answerer, mean wait time after questioning, reasons for not questioning, reasons for not waiting, and the effect of the questioning technique. Opinions on the importance of the questioning technique and the expected positive role of questioning were graded on a 5-level scale. The items of the expected positive role of the questioning technique were decided on 4 items regarding the advantages mentioned in the literature [2,3,7-10]. In addition, questions on whether to plan for questioning (prepare before class, question impromptu during class, not question), questioning time (in the introduction of the class, in the middle of the class, at the end of the class), type of questions (closed, open), intended answerer (a specific person, the entire group, and if no response, a specific person) were posed so that the sum of the full scores for the items was 100 %.

The observation records, which were for understanding the characteristics of questioning during class, included data, such as the date of observation, class name, faculty name, total class time, whether to use questioning, question type, intended answerer, whether to wait after questioning, wait time, whether students answered or not, and the faculty members’ response to the students’ silence. There are two types of wait times [12]. Wait time 1 is the time a teacher pauses after asking a question and calling upon a student to answer. Wait time 2 is the time from after a student completes an answer to when the teacher resumes the presentation or asks another question. Wait time 1 was evaluated in this study and the term wait time in this article means wait time 1.

### Data collection

Data collection using a questionnaire was performed by 2 researchers (MJB, YHY). The researchers visited each faculty, explained the purpose of the study, distributed questionnaires, and collected them when they were filled out. A total of 40 questionnaires were distributed, of which 33 (82.5 %) were completed. Among the 7 faculty members from whom the questionnaires were not obtained, 3 refused to answer and 4 gave incomplete responses. In data collection through observation, videos of the classes recorded using a camcorder (Sony, HDR-HC3) from September 8 to November 14, 2008 were analyzed, and observation record sheets were filled out. Thirty three faculty members who completed the questionnaires were included in this study. Video was analyzed independently by 2 teams and each team was consisted of two observers. Four researchers (YHC, DWJ, SJI and JGL) received an education about the analysis methods from a supervisor (SYL). The supervisor confirmed the results if there was disagreement between observations of the video record. The researchers did not know the rank of the faculty member. The wait time was measured manually using a stop watch, and the observation records of 99 classes lectured by 33 faculty members were analyzed.

### Ethical review

The study was approved by the Institutional Review Board at Pusan National University Hospital. Informed written consent was obtained from each faculty member and student prior to video recording.

### Analysis

The data was analyzed statistically using SPSS 14.0 for Windows (SPSS, Chicago, IL, USA). Data were analyzed only if both survey research and observation research were done. When a faculty had more than one class, mean value is used as representative value of the participant. The data distribution was tested for normality with the D' Agostino-Pearson test. Items showing a regular distribution are presented as the mean and standard deviation, whereas those not showing a regular distribution are reported as the median and range. A total of 99 classes were analyzed, and after excluding 40 without a questionnaire, the questioning technique was analyzed again using 59 classes that asked one or more questions.

Inter-observer team agreements were excellent for measurement of the wait time after questioning (kappa = 0.92). Survey of faculties’ questioning technique and observation results were described by mean percentage and standard deviation. A Wilcoxon signed-rank test was used to compare the wait time after questioning perceived by the participants with the actual wait time, and a Kruskal-Wallis test was used to compare the results according to the faculty position.

## Results

### Demographic characteristics of participants

Of the 33 participants, 27 (81.8 %) were male, and their mean age was 45.2 ± 8.8. According to the organization, 26 (78.8 %) were faculties from clinical departments and 7 (21.2 %) were faculties from basic medical departments. The mean teaching experience was 9 (0.5-29) years and the participants were 33.3 percent of assistant professors, 36.4 percent of associate professors, and 30.3 percent of full professors according to their faculty position (Table 1).

**Table 1:** Demographic characteristics (N = 33)

**Characteristic**	**Number**	**%**
Sex		
Male	27	81.8
Female	6	18.2
Department		
Clinical	26	78.8
Basic medical	7	21.2
Position of faculty		
Full Professor	10	30.3
Associate Professor	12	36.4
Assistant Professor1030.3	11	33.3
Age ^*^		45.2 ± 8.8

^*^Age is shown as mean age ± standard deviation.

### Faculty members’ perception of the questioning technique

With regard to the importance of questioning technique during classes, 28 subjects (84.8 %) regarded it as important, and with regard to the expected effects of questioning technique, over the half of participants replied that it performs positive functions for students’ participation in class, concentration on class, and understanding of class contents, showing that university faculties generally perceive questioning technique to be effective. To the question of whether questioning technique creates a comfortable atmosphere for students to ask questions, however, 16 (48.5 %) participants replied ‘So-so’ (Table 2).

**Table 2:** Importance of the questioning technique during classes and the expected effects of the questioning technique

**Description**	**Item**	**Number**	**%**
Importance of questioning technique during classes			
	Very important,	10	30.3
	Important	18	54.5
	So-so	5	15.2
	Not important	0	0.0
	Not important at all	0	0.0
Positive functions for students’ participation in class			
	Very positive	10	30.3
	Positive	21	63.6
	So-so	2	6.1
	Not positive	0	0.0
	Not positive at all	0	0.0
Concentration on class			
	Very positive	8	24.2
	Positive	22	66.7
	So-so	3	9.1
	Not positive	0	0.0
	Not positive at all	0	0.0
Understanding of class contents			
	Very positive	11	33.3
	Positive	15	45.5
	So-so	7	21.2
	Not positive	0	0.0
	Not positive at all	0	0.0
Creating a comfortable atmosphere for students to ask questions			
	Very positive	1	3.0
	Positive	14	42.4
	So-so	16	48.5
	Not positive	2	6.1
	Not positive at all	0	0.0

As to planning for questioning, participants reported that 33.6 % of them prepared questions in advance and 41.6 % questioned impromptu without preparation. As to the time of questioning, most of faculties answered they used questioning in the middle of class. As to intended answerer, 54.8 % of participants replied that they intended to use questioning to the entire group. As to question type, faculties reported that they asked open type of questions (66.7 %) twice more than closed type of questions (33.3 %, Table 3).

**Table 3:** Faculty members’ perception of the questioning technique

**Description**	**Item**	**Mean percentage (%)**	**SD**
Planning questions			
	Prepare in advance	33.6	33.1
	Questioning impromptu without preparation	41.6	31.9
	No questioning	24.8	30.2
Time of questioning^*^			
	In the introduction of class	16.3	19.7
	In the middle of class	67.0	30.4
	At the end of class	16.7	21.0
Intended answerer			
	Individual	22.7	27.5
	Entire group	54.8	35.6
	Entire group → individual^§^	22.4	26.5
Type of question			
	Open questions	66.7	24.8
	Closed questions	33.3	24.8

SD: standard deviation.

^*^ In the introduction of class, first 20 % of the total class hour in the middle of the class, in the middle of 20 %-80 % of the total class hour; at the conclusion of class, the last part of the class after 80 % of the total class hour.

^§^if there was no answer from the questioned group, the question can be given to individuals.

The perceived number of questions per class was 2 (0–50), and the perceived wait time after questioning was 10 (3–60) seconds. More than 50 % of participants reported a lack of time as the reason for not questioning or not waiting after questioning.

### Actual questioning behaviors

In the 99 classes analyzed, the mean class time was 53.4 (14–127.2) minutes, and the median number of questions per class was 1 (0–29). Among them, 40 classes (40.4 %) did not use a questioning technique at all. Among 33 professors, also, 4 of them never used questioning technique. As to questioning behaviors in actual classes analyzed using videos that recorded the classes, the most frequent questioning timing by faculties was introduction of class, contrary to their perception. In case of intended answerer, the faculties asked questions mainly to the entire group of students more than they perceived. In actual class, 69.4 % of questions were open questions, which was not different from perception of faculties (Tables 3 &4).

**Table 4:** Questioning behaviors in actual classes

**Descriptions**	**Items**	**Percent (%)**	**SD**
Time of questioning			
	In the introduction of class	42.2	40.7
	In the middle of class	24.5	29.2
	At the end of class	33.3	39.7
Intended answerer			
	Individual	20.9	34.1
	Entire group	76.3	35.7
	Entire group → individual^§^	2.8	13.9
Type of questions			
	Open questions	69.4	39.3
	Closed questions	30.6	39.3
Answering by students			
	Answered	33.6	38.6
	No answer	66.4	38.6

SD: standard deviation.

^*^ In the introduction of class, first 20 % of the total class hour, in the middle of the class, in the middle of 20 %-80 % of the total class hour; at the conclusion of class, the last part of the class after 80 % of the total class hour.

^§^if there was no answer from the questioned group, the question can be given to individuals.

### Difference between the faculty members’ expected questioning technique and their actual technique

**Figure 1:**
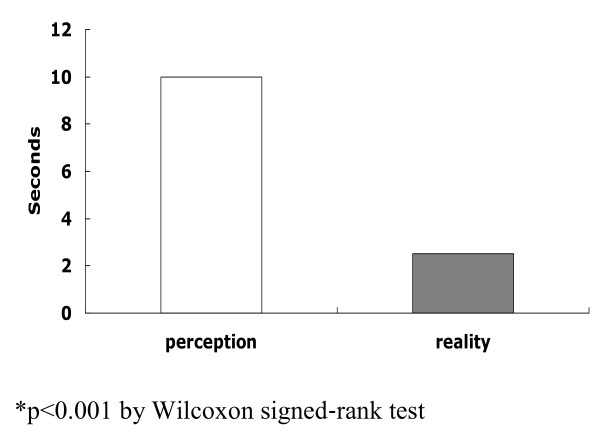
Difference between faculty members’ expected wait time and their actual wait time.

The actual wait time after questioning was 2.5 seconds, which was significantly different from expected wait time of 10 seconds (Figure 1, p < 0.001 by Wilcoxon signed-rank test). The wait time was similar regardless of the faculty members’ position, but the number of questions per class increased in the order of assistant professors, associate professors and professor (Table 5).

**Table 5:** Number of questions and expected wait time according to faculty members’ position

**Category**	**Assistant professor (n = 11)**	**Associate professor (n = 12)**	**Full professor (n = 10)**	p-value^*^
No. of questions per class	1.5 (1.0-20.0)	3.0 (1.0-10.0)	6.0 (1.0-29.0)	0.049
Expected wait time	10.0 (3.0-20.0)	10.0 (3.0-60.0)	5.0 (0.0-10.0)	0.062
Actual wait time	1.7 (0.4-12.7)	2.9 (0.3-12.4)	2.5 (0.2-23.4)	0.278

* p value are based on comparisons using Kruskal-Wallis Test.

## Discussion

The results of the present study indicate that faculty members place a high value on the questioning technique in medical classes but their actual question applications were insufficient to show an effect on improving learning. Of the participants of this study, 85 % were aware of the importance of the questioning technique, and perceived that the questioning technique had positive influences on the students’ participation in class, concentration in class, and understanding of the class contents, but only 34 % of classes, question was prepared in advance before class. Among 99 classes, 40 (40.4 %) did not use a questioning technique at all. Therefore, it is necessary to plan questions before class and use the questioning technique adequately in each class.

When faculty members employed the questioning technique, the actual wait time after questioning was 2.5 seconds, which was much different from their awareness (Figure 1). Duell et al. [6] examined the wait time in college classes and found that the mean faculty members’ wait time 1 was 2.25 seconds, which was similar to the actual wait time in the present study. This was desirable compared to the 0.9 seconds reported for elementary and secondary teachers [4,5] in elementary school. On the other hand, one study reported that waiting for 3-5 seconds after questioning increases the mean length of the students’ answers, encourages voluntary and adequate answers, reduces the number of failed answers, promotes speculative answers, increases the students’ questions, and improves the students’ academic achievement [4]. Extending the wait time after questioning to 10–15 seconds is considered desirable [13] particularly because many questions posed in medical school demand high-level thinking [14]. An additional questionnaire on the ideal number of questions per class and wait time was given to 100 students attending classes. The median ideal number of questions per class and median ideal wait time was 5 (1–20) and 12.5 (3–60) seconds, respectively. For these reasons, it is necessary to increase the wait time. Faculty members may be able to wait 10-15 seconds after questioning if they count the wait time after questioning or perform slow breathing three times. A lack of time was the most frequent reason for not questioning during classes and not waiting for a reply. As suggested by the explanation that questions provide students with clues to the contents and directions of learning [15], faculties may expect indirect learning effects from questioning. Therefore, they need to plan a part of class time as questioning time.

Open questions extract students’ thinking by inducing expansive thought but closed questions are not as stimulating as open ones [16], so open questions are recommended for questioning in class. Fortunately, in the present questionnaire survey, most of those who used questioning replied that they used open questions, and in reality, open questions were used frequently. The number of questions per class increased in the order of assistant professors, associate professors, and professors. This suggests that faculty members experienced in education spend a longer time in questions during their classes. Therefore, it is necessary to educate faculty of the importance of the questioning technique and the use of a questioning technique in class for those at the initial stages of their teaching career.

Amin and Khoo [13] emphasized the questioning technique in medical education and mentioned that good questions during class help the students to participate actively in lectures, and provide an opportunity to students to express their thoughts. In addition, they explained the faculty members needed to consciously practice simple questioning techniques. Craig and Page’s study [17] required teachers to complete a self-instructional module referring to the different levels of questioning, the importance of asking higher level questions and how to ask them. They found that the questioning ability can be improved when the teachers were taught about the levels of questioning and the importance of higher level questions.

There were some limitations to this study. First, this study was limited to some classes during a semester of a medical school and to faculty members who gave their consent to video recording, so the results cannot be generalized. Second, this study divided the total class time of a class into three parts, that is, the first 20 %, middle 60 %, and last 20 %. In general, a class is divided into three stages, namely, introduction, development and conclusion, but most medical school classes do not have such stages because each class should cover a large volume of contents. Therefore, although the first 20 % of the total class time may be the early part, it may not be exactly the introductory stage of the class. Third, this study analyzed only wait time 1 after questioning until students answered the question using video records. Previous studies reported that wait time 2 after students have finished answering a question until the faculty resumed the class or asked another question is important for enhancing the effect of the questioning technique [5]. Fourth, each question was not classified into categories according to the purpose in the present study. Rhetorical questions can be used for good or bad purposes. There was also a limitation with the possible underestimation of the wait time. On the other hand, more than half of the participants mentioned a lack of time as the only reason for waiting after questioning. Finally, as this study focused on questioning among the faculty members’ linguistic response during classes, it did not examine the interaction between faculty members and students. Further research considering wait time 2 and the cognitive level of the questions in medical class will be needed to extend our findings. In addition, it will be also necessary to examine the impact of faculty development programs on the effective questioning skills of medical educators.

## Conclusions

Few studies have examined the faculty members’ perceived questioning technique during classes and their real questioning technique in medical schools. This study analyzed class video recordings. Therefore, this study is meaningful in that it made objective analysis of the questioning behaviors used in medical school classes.

There were some discrepancies regarding the use of a questioning technique between the faculty members' perceptions and reality, even though both faculty members and students had positive opinions using the technique. Accordingly, questioning skills during a lecture need to be emphasized to faculty members.

## Competing interest

The authors declare no competing interests.

## Authors’ contribution

YHC, SYL, SYB and YJK were responsible for the study concept and design. MJB and YHY contributed to data collection using the questionnaire from faculty members. YHC, DWJ, SJI and JGL analyzed video observations. SYL supervised the video observations analysis. SYL, YJK and JGL analyzed the data statistically. EJC, SHL and SJY contributed to the analysis with suggestions and advice. YHC prepared the manuscript. All authors read and approved the final manuscript.

## Pre-publication history

The pre-publication history for this paper can be accessed here:

http://www.biomedcentral.com/1472-6920/12/39/prepub
